# Understanding variations in the use of tranexamic acid in surgery: A qualitative interview study

**DOI:** 10.1111/bjh.20008

**Published:** 2025-02-18

**Authors:** Louise Strickland, Hayley G. Evans, Antony Palmer, Samantha Warnakulasuriya, Michael F. Murphy, Simon J. Stanworth, Robbie Foy, NIHR BTRU in data driven transfusion practice collaborators

**Affiliations:** ^1^ Nursing and Midwifery Research and Innovation and Honorary Departmental Clinical Academic Nurse Researcher Oxford University Hospitals NHS Foundation Trust and Nuffield Department of Orthopaedics, Rheumatology and Musculoskeletal Sciences (NDORMS) University of Oxford Oxford UK; ^2^ NIHR Blood and Transplant Research Unit in Data Driven Transfusion Practice, Nuffield Division of Clinical Laboratory Sciences, Radcliffe Department of Medicine University of Oxford Oxford UK; ^3^ Oxford University Hospitals NHS Foundation Trust and Nuffield Department of Orthopaedics, Rheumatology and Musculoskeletal Sciences (NDORMS) University of Oxford Oxford UK; ^4^ Anaesthesia and Perioperative Medicine University College London Hospital NHS Foundation Trust London UK; ^5^ Transfusion Medicine at the University of Oxford and Consultant Haematologist for NHS Blood & Transplant (NHSBT) and Oxford University Hospitals NHS Foundation Trust Oxford UK; ^6^ NHSBT Oxford University Hospitals Foundation Trust and Professor of Haematology and Transfusion Medicine at the University of Oxford Oxford UK; ^7^ Primary Care, Leeds Institute of Health Sciences University of Leeds Leeds UK

**Keywords:** major blood loss, surgical practice, tranexamic acid

## Abstract

Despite robust supporting evidence, around a third of eligible surgical patients do not receive tranexamic acid (TXA). Effective strategies based on an understanding of clinical behaviour are needed to increase use and improve patient outcomes. We conducted semi‐structured interviews with clinicians involved in perioperative care to explore perceived influences on TXA use. We identified key influences on practice using the theoretical domains framework. We matched these to behaviour change techniques and evidence‐informed implementation intervention components. Across 22 interviews, we identified eight key influences within three overarching themes of capability, opportunity and motivation. Capability influences included the clinical context and variable familiarity with TXA. Opportunity concerned the availability of both TXA and checklists to support decision‐making and whether TXA use was consistent with professional expectations and perceived responsibilities. Motivation concerned confidence in administering TXA, perceived benefits and risks and training received around potential risk factors. These influences varied across participants and specialities. Our resulting proposed implementation strategy included training, clinical prompts, comparative performance feedback and opinion leadership supported by specialty‐specific guidance. Any strategy to increase TXA use that improves knowledge and skills without addressing wider influences on clinical behaviour is only likely to meet with limited success.

## Graphical abstract

The use of tranexamic acid (TXA) to reduce surgical bleeding is highly variable with scope for improvement. Effective implementation strategies should be based on an understanding of clinical behaviour. We interviewed a range of clinicians involved in perioperative care to elicit key influences on TXA use. These included the clinical context, variable familiarity with TXA, the availability of both TXA and checklists to support decision‐making, professional expectations and perceived responsibilities, confidence in administering TXA and perceived benefits and risks. These influences, which varied across participants and specialities, offer targets for coordinated implementation strategy.

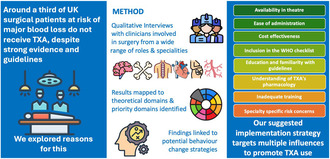

## INTRODUCTION

Blood transfusion services are struggling to meet current demand for blood transfusion, and blood shortages increasingly occur in resource‐rich^1^, ^2^ and resource‐poor countries.^3^ There is a need for mitigation strategies to reduce the need for transfusion. Surgery remains a significant indication for blood transfusion, and tranexamic acid (TXA) can safely reduce blood loss and the need for transfusion.^4^, ^5^, ^6^

However, there is highly variable use of TXA in perioperative practice^7^ A national audit in 2023 found that, in the United Kingdom, a third of eligible surgical patients were not given TXA.^8^, ^9^ Therefore, up to 500 000 out of the 1.5 million people undergoing major surgery each year in the United Kingdom may not receive TXA.^10^ Optimal use of TXA could result in 147 fewer blood transfusions per 1000 people^6^ and significantly reduce hospital stays.^11^ TXA has a key role in reducing potential harms such as exposure to blood transfusion, delayed hospital discharge and unplanned readmissions.^12^ In addition to longer term compromised health and recovery associated with anaemia^13^ Given the relatively modest cost of TXA,^14^ there is also a strong economic case for greater use.

Any coherent and effective strategy to close this gap between evidence and practice requires an understanding of the salient influences on clinical behaviour which are amenable to change. We therefore aimed to identify key influences on the use of TXA in surgery and develop an implementation strategy to promote more rapid and equitable uptake.

## METHODS

### Design

This qualitative semi‐structured interview study drew on the theoretical domains framework (TDF) to explore influences on perioperative prescribing and administration of TXA. The TDF was designed for implementation research to identify determinants of health professional behaviours^15^, ^16^ In synthesising 33 behaviour and behaviour change theories into key domains, it provides a theoretical perspective on cognitive, affective, social and environmental influences on clinical practice. We followed suggested methods for conducting a TDF‐informed interview study.^17^ We discussed our plans and shared emerging findings through regular patient and public involvement panel meetings, to help contextualise and frame our results.

### Sample and setting

The study targeted clinicians from a range of roles and specialities involved in decision‐making, prescribing or administering TXA at Oxford University Hospitals NHS Foundation Trust. We anticipated reaching data saturation, where no new themes emerge, after 20–30 interviews.^18^ All participants were recruited through professional networks and provided informed consent. Ethical approval was granted by the Medical Sciences Interdivisional Research Ethics Committee (MS IDREC) Number: R85942/RE001.

### Interview procedure

A multidisciplinary team, with experience in transfusion medicine and implementation research, developed the interview guide. The guide was pretested with four clinicians to ensure comprehensiveness and acceptability and refined prior to study interviews (Table S1). We initially asked participants about their professional backgrounds, roles and specific practices concerning presurgical risk stratification for blood loss and TXA use. Interviews were conducted face to face, by videoconferencing or by telephone as participants preferred.

### Data analysis

Initial coding involved familiarisation with transcripts and developing a coding framework, employing both inductive and deductive logic informed by the TDF and research goal. To avoid overlooking overarching themes, researchers reviewed each transcript before coding. Initial coding was performed by HE and LS, who met regularly with RF to promote consistency and capture of key data categories.

We applied the framework to the complete dataset, involving indexing and charting data into the TDF domains. We undertook comprehensive coding of the dataset, compiling coded data fragments across cases within the TDF domains. We used *NVivo* software to facilitate the application of the coding framework and compilation of codes, ensuring transparency by linking codes to source data.^19^ We (HE and LS) met early in this process to achieve consensus on code application, independently coding 10% of interviews and resolving disagreements through discussion.

We (HE and LS) conducted the initial TDF analysis, examining data within each domain and creating tables highlighting key thematic content. A thematic analysis within each domain identified unique influences, with researchers independently prioritising primary TDF domains for each item. We resolved disagreements through discussion with a third researcher (RF). The broader research team reviewed tables linking domains with sample data to ensure diverse perspectives were considered in interpreting the findings.

We (RF, HE and LS) independently reviewed the summarised data, reaching a consensus to identify a shortlist of the most significant domains influencing TXA utilisation, based on the frequency and richness of content.

We further grouped domains using the COM‐B model of behaviour change.^20^ This proposes that, to engage in a behaviour, a person must have the physical and psychological capability (C) and opportunity (O) to exhibit the behaviour, as well as the motivation (M) to demonstrate the behaviour at that specific moment.

We matched identified theoretical domains to potentially relevant behaviour change techniques using the theory and technique tool.^21^ We drew upon group judgement, as well as behavioural change theory and evidence, in considering relevant behaviour change techniques. We then considered and proposed implementation intervention components, drawing upon summarised evidence from systematic reviews^22^ and judgements about likely feasibility and acceptability.

## RESULTS

We interviewed 22 clinicians (14 male, 8 female) working in surgical settings (Table 1). Nine self‐identified as White British; three as Black, African, Caribbean or Black British; two as Asian or Asian British; and eight as other ethnic groups. Interviewees comprised 12 surgeons, seven anaesthetists and three anaesthetic and cell salvage practitioners (registered nurse and operating department practitioners). Nine worked in orthopaedics (including trauma, spinal, sarcoma and arthroplasty), three in general surgery, three in vascular surgery, three in neurosurgery, two in obstetrics and two in cardiac surgery. Twenty‐one had worked up to 20 years since graduation and one for over 40 years.

**TABLE 1:** Participant characteristics.

Participant characteristics	
Number of participants	22
Age range (years)	
35–44	7
45–54	9
Over 55	6
Gender	
Male	14
Female	8
Ethnicity	
White British	9
Asian/Asian British	2
Black/African/Caribbean/Black British	3
Other ethnic group	8
Employment Role	
Surgeons Including specialists in hip and knee, paediatric deformity, complex adult spine, obstetrics and foetal medicine, hepato‐biliary, obstetrics, neurosurgeon x2, shoulder and upper limb, colorectal fellow, trauma and vascular fellow, vascular trainee	12 (9 consultants, 3 trainees)
Anaesthetic consultants Including orthopaedics and neurosurgery, cardiac and orthopaedics, ITU, vascular and plastics, cardiac and thoracic, spinal sarcoma and orthopaedics, intensivist, vascular and hepatobiliary	7
Anaesthetic and cell salvage practitioners Including registered nurse and operating department practitioner	3
Length of time in career (years)	
0–10	9
10 to 20	12
21–30	0
Over 40	1

**FIGURE 1:**
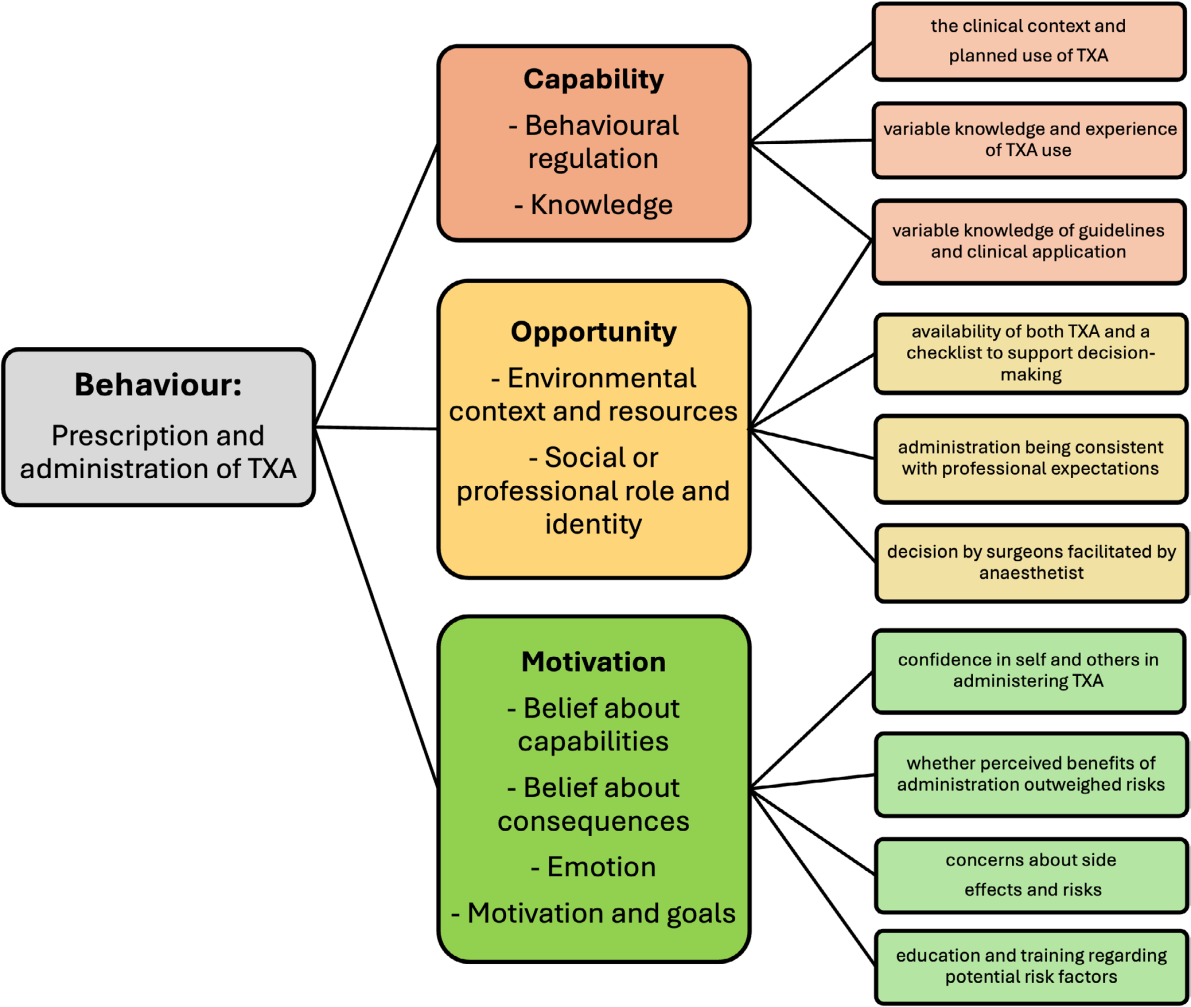
Influencers of TXA usage.

We coded a total of 1066 utterances into 10 theoretical domains. Eight key domains emerged as major influences on the pre‐operative prescription and administration of TXA in cases where moderate blood loss (>500 mL) was anticipated (summarised in Table 2 and presented in full in Table S2). We grouped these domains under capability (behavioural regulation and knowledge), opportunity (environmental context and resources and social or professional role and identity) and motivation (belief about capabilities, belief about consequences and emotion), summarised in Figure 1.

**TABLE 2:** Summary of domains and illustrative quotes by theoretical domain.

Domain	Example quotes
Capability	
Behavioural regulation	‘I use it on every single patient that I'm expecting major blood loss’ (Pt1, anaesthetist)
	‘And most of the vascular surgeons do not administer TXA, even though, you know, we're ticking dutifully on the form that there is a risk of more than 500 mLs blood loss’ (Pt10, anaesthetist)
Knowledge	‘You know it's just a drug something to do with clotting. There's a there's a sort of a misunderstanding that it forms clots and enables clots to form, which isn't what it does at all’ (Pt5, cell salvage practitioner)
	‘I've attended many anaesthesia conferences, many regular anaesthesia conferences over the last sort of three to five years and TXA has routinely come up in these conferences’ (Pt22, anaesthetist)
	‘I would say I have not been trained in its utilisation at all’ (Pt7, surgeon)
	‘I think yes. I think there is enough communication and dissemination of the information’ (Pt12, cell salvage practitioner)
Skills	‘So our standard guidelines is if (they are) bleeding and has bled more than a litre than we should think about TXA…But it's usually, you know, because it's such a standard part of our protocol, it's just usually ‘oh, (they are) now bleeding. Please, can you give some TXA, please?” (Pt8, surgeon)
	‘I think I'm probably influenced by my experience and my previous kind of working with teams where I think you know, there's a dogma is a bit of a kind of influence, influential word’ (Pt18, surgeon)
	‘So maybe having some education and particularly evidence and potential risk factors would help’ (Pt16, surgeon)
Opportunity	
Environmental context and resources	‘Yeah. I haven't come across this situation where it hasn't been available to date’ (Pt16, surgeon)
	‘I would give it even though I'm expecting less than 500mls because it is an easy‐to‐use drug, safe and is cheap’ (Pt1, anaesthetist)
	‘That's a key question that I think sometimes gets lost in the lack of sensitivity of these checklists or this individual patient in front of you’ (Pt10, anaesthetist)
	‘The TXA question became part of the WHO procedure. So that made everyone realise, well, apparently for all the specialities it is a completely normal thing to include in your daily practice’ (pt17, surgeon)
	‘Our orthopaedic surgeons are tuned into it, spinal surgeons are tuned into it. And other areas are less so… if it's a relatively small operation…then asking about TXA, it's…not relevant’ (Pt3, anaesthetist)
Social and professional role and identity	‘In the morning meeting, we have a safety checklist, WHO checklist, and as part of that the anaesthetist for pretty much every case I do will be asked to give antibiotics and generally asked to give TXA’ (Pt15, surgeon)
	‘Yes, that is something that at the beginning it is discussed’ (Pt4, cell salvage practitioner)
	I think it's the consultant surgeon (Pt2, surgeon)
	The final decision will be from the surgeon or anaesthetic team, but it will mostly be the anaesthetist that gives it (Pt4, cell salvage practitioner)
	When I started, nobody gave it. So, I started doing anaesthetics in 2000. So, nobody gave it back then. And then when it was a trainee. And it used to be cardiac only (Pt3, anaesthetist)
	‘Probably fairly nebulous decisions… really just on the difficulty of the surgery’ (Pt7, surgeon)
	‘I think the difficulty is judging accurately where the risks exceed the benefits, and it would be nice to see where the benefits exceed the risks in those borderline cases’ (Pt9, surgeon)
	‘The vascular surgeons are not that keen on trying tranexamic acid yet… because we have cell salvage for their cases, it's not thought of as needed’ (Pt22, anaesthetist)
Motivation	
Beliefs about capabilities	‘I would say we're very confident’ (Pt6, surgeon)
	‘I think we're all in the same boat. We are a little bit uncertain’ (Pt9, surgeon)
	‘I think that now it has become more common for my colleagues to use TXA not only in spine surgery and because it is part of the protocol in knee and hip surgery, but also now it is widely used for head and neck surgery’ (Pt19, surgeon)
	‘The anaesthetists feel very strongly. It shouldn't be given until the woman's haemorrhaged, and I agree with that because that's how it was used in the study’ (Pt11, surgeon)
Beliefs about consequences	‘I don't believe it has any risk and I think it's a good drug with benefits’ (Pt5, cell salvage practitioner)
	‘I suppose the risk, well the risk profile in my mind, correctly or wrongly (laughs), is those patients who are at higher risk of thromboembolic events. There might be a risk in using it there’ (Pt2, surgeon)
	‘I mean, I think for in the cardiac surgical setting, the benefits definitely outweigh the risks’ (Pt13, anaesthetist)
	‘So, I personally I'm not aware of a complication that we have attributed directly to TXA’ (Pt10, anaesthetist).
	‘We've been using it for quite a while so you know how much difference does it make? I don't really know. Because we always use it and therefore we don't really notice whether it reduces blood loss or not. We just assume that it does (help)’ (Pt6, surgeon)
Emotion	‘Nothing. It's just like using local anaesthetic or antibiotics. It's just something that you give’ (Pt6, surgeon)
	‘it feels like a very benign drug’ (Pt11, surgeon)
	‘if it goes wrong, there is such a huge impact on the patients…So, what is the right thing in which situation?’ (Pt20, surgeon)
	‘And when I've heard the surgeons discuss it, it's fear rather than evidence. Or even anecdotes. Anecdotal experience’ (Pt10, anaesthetist)
	‘I've known neurosurgeons to say no thanks cause of that worry. So, there's still worrying about thrombosis’ (Pt3, anaesthetist)
	‘my main concern with the big cases is that the long cases, 6, 7, 8 hours of operating, meaning that you know, if I'm worried about increasing the risk of thrombosis…there is in general a hesitancy of using it…I think that it's a very safe drug and I think we should be using it more and more… I'm getting there to be convinced for, you know, regular use, so I'm using it more. But you know we are very, very, very scared of having thrombotic events in patients because they are all you know, having problems with mobility and things like that’ (Pt14, surgeon)
	‘I think no one is really worried about giving it. It is just the more that they are not familiar and have not have never used it before in their twenty year long career, so they, they feel reluctant of suddenly doing something new’ (Pt17, surgeon)
	‘I think there's a there's a reluctance almost to give something that people may have this fear of causing thrombosis and graft failure in vascular. That's kind of that's almost an own goal for us’ (Pt18, surgeon)
Motivation and goals	‘I think there needs to be there is likely to be a period of five or ten years. I suspect where it's true role is actually found. And as I've said, you know most of the tranexamic acid I'm giving, I think is in non‐evidence domains and I'm not entirely sure that people are actually genuinely trying to find the evidence for it's benefit. Or for its potential harms in those contexts’ (Pt10, anaesthetist)
	‘So maybe having some education and particularly evidence and potential risk factors would help’ (Pt16, surgeon)
	‘Well, I think even I, even though I blood loss is minimal, I mean if it's 200 and you can bring it down to 100 and I think it's still of value’ (Pt17, surgeon)
	‘I guess so. People tend to fall into being users or not seem to bother with it. All the surgeons I work with are very keen on it’ (Pt1, anaesthetist)
	‘I mean we all know that if you attach a financial benefit to doing something in any trust that's switched on, well we will endeavour to comply’ (Pt6, surgeon)
	‘It's probably not because I think it probably is being used appropriately and in the correct way and at the correct time in the majority of cases only because we're almost, as I said, too trigger happy with TXA now’ (Pt8, surgeon)
	‘It can be…The problem…is the teams actually doing the work don't get the benefit’ (Pt7, surgeon)
Social influences	‘Now you're having surgeons worried about their blood product usage has certainly focused their minds about TXA usage. We have regular audits and cardiac and orthopaedic surgeons are looking more and more blood product usage as a surrogate for quality of care’ (Pt1, anaesthetist)
	‘The blood shortage was also a driver for the increased use of TXA, as with any other patient blood management techniques…I think it's trying to find strategies to mitigate the short age of blood’ (Pt12, cell salvage practitioner)
	‘I think now it's part of our WHO checklist and it's asked every single time, every procedure and we're not entirely sure where this came from because I don't think it was actually the vascular surgeon involved in this. But it seems to be the Trust, you know, the WHO checklist we do at the beginning of every operation that seems to have changed…and suddenly there. ‘Do you want TXA as part of every question?’ Every time we discuss it and majority of us say no, you know’ (Pt20, surgeon)

### Capability

In considering *behavioural regulation*, clinical contexts and procedures play significant roles in TXA use. Some clinicians administer TXA routinely for patients expected to have major blood loss (Pt1) but reported practice varied by specialty. Specialities such as vascular surgery were less likely to administer TXA despite recognising the potential for significant blood loss (Pt10). Limited *knowledge* around TXA mechanisms of action was a prominent barrier (Pt5) and participants reflected on the absence of training to address this need (Pt12). Conference attendance was mentioned as a dissemination strategy (Pt22), but many participants had received little or no training on TXA administration (Pt7).

### Opportunity

In terms of *environmental context and resources*, all participants reported that TXA was consistently available, inexpensive (Pt1) and easy to administer (Pt16). TXA had recently been added to the WHO surgical safety checklists used across this trust. Some participants reflected that including TXA had helped emphasise routine use and normalised administration (Pt17) by promoting discussions ahead of surgery. Others criticised the checklist's lack of specificity (Pt10) and felt that administration depended on cultural norms within specific surgical teams (Pt3).

Participants universally acknowledged that recommending, discussing or prescribing TXA was part of their *social and professional role and identity*. They mentioned team discussions, typically during the World Health Organization (WHO) Checklist briefings, as crucial for deciding on TXA administration (Pt15, Pt4). Decision‐making was typically collaborative, involving both anaesthetists and surgeons. While the final decision to use TXA often rested with the surgeon, anaesthetists were primarily responsible for administration (Pt2, Pt4) and their advocacy sometimes gradually led to it becoming standard practice (Pt3). Participants felt that the criteria for prescribing TXA could be unclear (Pt7), presenting a challenge of assessing risks versus benefits, especially in borderline cases (Pt9). Specifically, vascular surgeons were less inclined to use TXA given concerns over clotting risks (Pt18) and the availability of cell salvage (Pt22).

### Motivation

When considering *beliefs about capabilities*, participants exhibited varying levels of confidence regarding the administration of TXA. Some expressed high confidence (Pt6) while others, particularly in cases involving chemotherapy, were uncertain (Pt9). Confidence was generally higher in cases of anticipated high blood loss, with increasing usage noted across various specialities (Pt19). However, confidence was conditional in obstetrics, with a preference to administer TXA only after significant blood loss had occurred (Pt11).

Participants held diverse *beliefs about the consequences* of TXA use. Direct experience with the routine use of TXA helped change beliefs and practice (Pt5). Some viewed it as a safe and beneficial drug (Pt5), while others expressed uncertainty about potential risks, particularly thromboembolic events (Pt2). Despite this, most participants believed that benefits outweighed the risks, especially in cardiac surgery (Pt13). No participant could recall any complications directly attributable to TXA administration (Pt10). Earlier career contributors already familiar with routine use of TXA could not make any judgements about benefits for individual patients (Pt6).

*Emotional* responses varied widely. Some participants viewed TXA as a routine, low‐risk drug, comparable to local anaesthetics or antibiotics (Pt6, Pt11). Others expressed apprehension due to uncertainty about potential risks, which could influence decision‐making (Pt20, Pt3). Despite these concerns, there was a desire to increase TXA use, particularly in longer or high‐risk surgeries (Pt14). Reluctance to adopt TXA was attributed to lack of familiarity among experienced surgeons (Pt17) or misinformation about risks (Pt18).

In terms of *motivation and goals*, there was a desire for further evidence and clinical experience to better define the appropriate use of TXA (Pt10). Participants suggested that education about TXA benefits and risks would motivate its use (Pt16). Highlighting its effectiveness in reducing blood loss, even in minor procedures, could encourage use (Pt17). While some participants felt that financial incentives could promote TXA use (Pt1, Pt6), others considered this approach as potentially controversial and inappropriate (Pt8, Pt7).

### Further relevant domains

Familiarity with specialty guidelines facilitated *skills* for TXA administration when embedded in protocols for managing significant blood loss (Pt8). While some participants gained confidence from personal or observed TXA use, others were discouraged by inexperience and resistance from senior colleagues (Pt18). Concerning *social influences*, TXA use had not become routinely established in specialities such as vascular surgery, despite organisational requests to consider it (Pt20). Blood shortages prompted use of TXA, reinforced by audits and increased awareness among surgeons of appropriate blood product use (Pt1, Pt12).

### Development of implementation strategy

In matching to behaviour change techniques, we included domains identified as key (i.e. knowledge, environmental context and resources; social or professional role and identity; beliefs about capabilities; beliefs about consequences; emotion; and motivation and goals) and others (skills and social influence). We included several matched behaviour change techniques where behavioural evidence was inconclusive but potentially relevant in this context, for example, social comparison for social or professional role and identity. Our resulting prototypical implementation strategy included specialty‐specific guidance, educational events and materials, simulation training, prompts and reminders, comparative performance feedback, modelling of behaviour by opinion leaders and collaborative problem‐solving (Table 3).

**TABLE 3:** TXA implementation strategy based on behavioural change theory and evidence.^a^

Influences on practice identified from interviews	Matched behaviour change techniques (‘active ingredients’)	Possible intervention components (‘delivery mechanism’)
Capability		
Knowledge	Instruction on how to perform the behaviour: *Advise or agree on how to perform the behaviour* Information about antecedents: *Provide information about antecedents (*e.g. *social and environmental situations and events, emotions, cognitions) that reliably predict performance of the behaviour* Information about consequences: *Provide information (*e.g. *written, verbal, visual) about consequences of performing the behaviour*	Speciality‐specific guidance on TXA prescribing and administration (who, why, when, how) Educational events specifying above and evidence base on known benefits, harms and costs of TXA
Skills	Instruction on how to perform the behaviour Behavioural practice or rehearsal: *Prompt practice or rehearsal of the performance of the behaviour one or more times in a context or at a time when the performance may not be necessary, in order to increase habit and skill* Graded tasks: *Set easy‐to‐perform tasks, making them increasingly difficult, but achievable, until behaviour is performed*	Speciality‐specific guidance on TXA prescribing and administration (who, why, when, how) Use simulation‐based training to enhance practical skills in administering TXA Educational vignettes with worked examples around when and how to prescribe TXA, initially focusing on patient groups and clinical circumstances perceived as highest need or least risky
Opportunity		
Environmental context and resources	Social support (practical): *Advise on, arrange, or provide practical help (*e.g. *from colleagues) for performance of the behaviour* Prompts and cues: *Introduce or define environmental or social stimulus with the purpose of prompting or cueing the behaviour. The prompt or cue would normally occur at the time or place of performance* Restructuring the physical environment: *Change, or advise to change the physical environment in order to facilitate performance of the wanted behaviour or create barriers to the unwanted behaviour* Restructuring the social environment: *Change, or advise to change the social environment in order to facilitate performance of the wanted behaviour or create barriers to the unwanted behaviour* Adding objects to the environment: *Add objects to the environment in order to facilitate performance of the behaviour*	Prescribing and administration speciality‐specific packs for TXA with practical instructions Integration of guidance into the WHO surgical safety checklist Digital reminders and prompts, e.g. clinical decision support tools within electronic health records to remind clinicians of TXA protocols at point‐of‐care and automated prompts based on clinical triggers
Social or professional role and identity	Social support (unspecified): *Advise on, arrange or provide social support (*e.g. *from colleagues) or non‐contingent praise or reward for performance of the behaviour. It includes encouragement and counselling, but only when it is directed at the behaviour* Social comparison: *Draw attention to others' performance to allow comparison with the person's own performance* Credible source: *Present verbal or visual communication from a credible source in favour of or against the behaviour* Identity associated with changed behaviour: *Advise the person to construct a new self‐identity as someone who ‘used to engage’ with the unwanted behaviour*	Promote inter‐professional collaboration to discuss and standardise TXA use in multidisciplinary teams Feedback including comparisons of prescribing with higher performing peers within own specialty or hospital Local opinion leaders (ideally including people who have recently changed their own practice) Role modelling and mentoring from experienced clinicians and clinical leaders who regularly use TXA
Social influences	Social support Social comparison Information about others approval: *Provide information about what other people think about the behaviour. The information clarifies whether others will like, approve or disapprove of what the person is doing or will do* Social reward: *Arrange verbal or non‐verbal reward if and only if there has been effort and/or progress in performing the behaviour*	Feedback including comparisons of prescribing with higher performing peers within own specialty or hospital and across hospitals and specialty networks TXA discussions in team meetings or interdisciplinary rounds to promote shared responsibility and build cultural consistency
Motivation		
Beliefs about capabilities	Problem‐solving: *Analyse, or prompt the person to analyse, factors influencing the behaviour and generate or select strategies that include overcoming barriers and/or increasing facilitators* Instruction on how to perform the behaviour Demonstration of the behaviour: *Provide an observable sample of the performance of the behaviour, directly in person or indirectly* e.g. via *film, pictures, for the person to aspire to or imitate* Behavioural practice or rehearsal Graded tasks Verbal persuasion about capability: *Tell the person that they can successfully perform the wanted behaviour, arguing against self‐doubts and asserting that they can and will succeed* Focus on past success: *Advise to think about or list previous successes in performing the behaviour (or parts of it)*	Facilitated group discussions on how to change or reinforce local policy and practice, bringing together teams with known higher and lower levels of TXA use Educational vignettes with worked examples around when and how to prescribe TXA, initially focusing on patient groups and clinical circumstances perceived as highest need or least risky
Beliefs about consequences	Information about consequences Salience of consequences: *Use methods specifically designed to emphasise the consequences of performing the behaviour with the aim of making them more memorable (goes beyond informing about consequences)* Anticipated regret: *Induce or raise awareness of expectations of future regret about performance of the unwanted behaviour* Pros and cons: *Advise the person to identify and compare reasons for wanting (pros) and not wanting to (cons) change the behaviour* Comparative imagining of future outcomes: *Prompt or advise the imagining and comparing of future outcomes of changed* versus *unchanged behaviour* Material incentives (for behaviour and outcomes): *Inform that money, vouchers or other valued objects will be delivered if and only if there has been effort and/or progress in performing the behaviour and achieving desired outcomes* Rewards (for outcome): *Arrange for the delivery of a reward if and only if there has been effort and/or progress in achieving the behavioural outcome*	Educational events and materials illustrating benefits of TXA use in typical patients and harms of not using TXA Financial incentives for both TXA prescribing and reductions in peri‐or post‐operative haemorrhage ‘Prizes’ for clinical teams or hospitals demonstrating best recent changes in increasing use of TXA
Emotion	Anticipated regret Reduce negative emotions: *Advise on ways of reducing negative emotions to facilitate performance of the behaviour*	Educational events and materials illustrating benefits of TXA use in typical patients and harms of not using TXA
Motivation and goals	Goal setting (behaviour and outcome): *Set or agree on a goal defined in terms of the behaviour and outcome to be achieved* Review of behavioural goals: *Review behaviour goal(s) jointly with the person and consider modifying goal(s) or behaviour change strategy in light of achievement. This may lead to re‐setting the same goal, a small change in that goal or setting a new goal instead of (or in addition to) the first, or no change* Discrepancy between current behaviour and goal: *Draw attention to discrepancies between a person's current behaviour (in terms of the form, frequency, duration, or intensity of that behaviour) and the person's previously set outcome goals, behavioural goals or action plans* Review outcome goals: *Review outcome goal(s) jointly with the person and consider modifying goal(s) in light of achievement. This may lead to resetting the same goal, a small change in that goal or setting a new goal instead of, or in addition to the first* Information about consequences Incentives (for outcome): *Inform that a reward will be delivered if and only if there has been effort and/or progress in achieving the behavioural outcome*	Setting local goals for TXA use and reductions in peri‐ or post‐operative haemorrhage Align TXA use with broader institutional goals, e.g. reducing blood transfusion needs Performance feedback illustrating gaps between recommended, perceived and actual practice Inclusion in continuing professional development or annual reviews.

^a^ There is repetition of several intervention components to demonstrate linkage to influences on practice.

## DISCUSSION

Variations in TXA use in perioperative care are likely to be attributable to a range of potentially modifiable influences. These include knowledge and beliefs about benefits and risks of administering TXA as well as social and environmental influences on clinical behaviour. We found differences in interpretations of applicability of evidence and hesitancy in use among specialities and teams that may help explain wider variations beyond one hospital trust. For example, both vascular and colorectal oncology teams showed particular resistance, and this was also described in a recent national survey of patient blood management practices conducted with RAFT.^23^ However, our study also illustrates how an evidence‐ and theory‐informed implementation strategy can be tailored to target key influences on clinical practice.

Knowledge and beliefs varied about TXA use, critically whether clinicians perceived that benefits would outweigh risks. The principle of ‘first do no harm’ was evident, with some clinicians avoiding TXA despite published evidence of benefit. In contrast, other clinicians, particularly those involved in surgeries with expected major blood loss, reported routinely using TXA. These clinicians experienced in TXA use generally expressed confidence in its safety and effectiveness, particularly in settings where its use is well established such as cardiac surgery^24^ and fields specifically supported by trial evidence, such as obstetrics and gynaecology.^25^

Understanding and experience varied across specialities, with some clinicians apparently lacking knowledge of TXA mechanisms of action and recommended clinical indications. Concerns were expressed about potential harms in other specialities, such as clotting risks in vascular surgery. Low awareness of clinical evidence may also hinder TXA use in trauma and surgical settings^26^, ^27^, ^28^ used the TDF to investigate barriers and facilitators to prehospital use of tranexamic acid and similarly identified a lack of knowledge and experience with TXA as well as confusion and restrictions relating to the guidelines for TXA administration.

Surgical team policies may be influenced by prevailing specialty norms and values as well as by gaps in knowledge and different interpretations of clinical research findings.^29^ Therefore, education addressing knowledge gaps needs to be accompanied by specialty‐specific guidance and social influence approaches to challenge and shift norms. Contextual factors, such as blood shortages, and ongoing quality improvement initiatives, such as comparative audits, also offered further motivations for increasing TXA use. Within the United Kingdom, additional pressure for change has come from recommendations of the infected blood inquiry to reduce the risks of transfusion.^30^

Participants reported that TXA was generally readily available, cost‐effective and easy to administer, making it a practical choice in many surgical settings, even those in low‐income countries.^31^ We found that including TXA within the surgical safety checklist can prompt discussions around it's use, a finding supported by the recent work of Murphy & Warnakulasuriya.^27^ Adding TXA to the WHO guidelines for postpartum haemorrhage has prompted efforts to improve its availability.^32^ While some clinicians appreciated the checklist as a reminder and discussion point for TXA administration, others criticised its lack of specificity, arguing that it sometimes failed to capture the nuanced needs of individual patients or specific surgical situations. While surgical checklists represent a relatively simple and promising strategy for addressing surgical patient safety worldwide, their implementation requires staff education, resourcing and a receptive culture within surgical teams.^33^

We highlight three main study limitations. First, the single‐site setting may limit the generalisability of our findings. Nevertheless, we used a structured approach to eliciting influences which has been found to be helpful across many other targeted clinical behaviours and contexts^17^ and which is likely to have captured most of the relevant influences on TXA use. Second, self‐reported influences on clinical behaviour may not reflect actual influences. Further methods such as ethnographic observations may shed light on contextual influences (e.g. pressures of decision‐making within a clinical environment) that participants may be less able to verbalise. Third, we made group judgements concerning the likely feasibility and acceptability of suggested implementation intervention components; these require further validation and refining with a wider group of clinicians who would ultimately be targeted by the implementation strategy. However, it does provide a clear set of challenges which should be considered when formulating a future implementation strategy.

Our work builds upon other implementation strategies relating to TXA that have been piloted with varying success.^34^, ^35^, ^36^ An effective strategy to accelerate and reduce variations in the uptake of TXA is likely to require several features. First, it needs to consider and align efforts across multiple levels of healthcare systems (national, organisational, team and individual).^37^ Our prototypical implementation strategy recognises this and includes suggestions, such as leveraging comparative performance feedback, which is of known effectiveness^38^ and targets organisations and teams rather than placing excessive emphasis on individual clinician behaviour change. Second, given multiple barriers to implementation, the strategy needs to be multifaceted in combining different interventions with embedded behaviour change techniques to address various influences on clinical practice. Hence, we suggest a suite of complementary implementation interventions. For example, a ‘graded task’ approach addresses limitations in skills and confidence by setting easy‐to‐perform tasks, making them increasingly difficult, but achievable, until the desired clinical practice is achieved more routinely. For this, we suggest educational vignettes with worked examples around when and how to prescribe TXA, initially focusing on patient groups and clinical circumstances perceived as highest need or least risky. However, this could be complemented by measures to make the clinical environment more conducive to TXA use, such as providing prescribing and administration packs for TXA with practical instructions. Third, the strategy needs to be tailored to address the needs and expectations of different specialities. Hence, we suggest specialty‐specific guidance combined with social influence approaches using opinion leadership^39^ and role modelling, ideally including clinicians from targeted specialities who have recently changed their own practice.

We acknowledge uncertainties inherent in our suggested strategy. For example, financial incentives tend to be seen as a panacea for encouraging recommended clinical practice but need to be carefully designed to avoid unintended consequences.^40^ There are also likely to be issues around costs and feasibility. For example, our patient and public involvement panel suggested that patients should routinely be informed about and offered TXA before surgery. Systematic reviews indicate that sharing such information with patients can be effective in changing clinical practice^41^; however, the feasibility and acceptability of routinely informing patients about TXA within presurgical counselling and consent procedures is unclear.

In conclusion, we have demonstrated a range of influences on prescribing and administration of TXA for surgery, highlighting differences between specialities. Many of these influences are amenable to change. The next steps are to further develop our prototypical implementation strategy and evaluate its cost‐effectiveness using a rigorous study design.^42^

## AUTHOR CONTRIBUTIONS

All authors: Concept, methodology, manuscript editing and approval. HE, RF and LS: Data generation and analysis, manuscript writing, editing and approval. All authors read and approved the final manuscript.

## FUNDING INFORMATION

This publication is supported by the National Institute for Health and Care Research (NIHR) Blood and Transplant Research Unit in Data Driven Transfusion Practice (NIHR203334). The views expressed are those of the author(s) and not necessarily those of the NIHR or the Department of Health and Social Care.

## CONFLICT OF INTEREST STATEMENT

Robbie Foy receives institutional grant funding from the UK National Institute for Health and Care Research (NIHR) and chairs the Implementation Strategy Group for the UK National Institute for Health and Care Excellence (NICE). The other authors declare no conflicts of interest.

## ETHICS APPROVAL STATEMENT

Ethical approval was granted by the Medical Sciences Interdivisional Research Ethics Committee (MS IDREC) Number: R85942/RE001.

## PARTICIPANT CONSENT STATEMENT

Informed consent was obtained from all individual participants included in the study.

## PERMISSION TO REPRODUCE MATERIAL FROM OTHER SOURCES

This study does not include any material reproduced from other sources.

## Supporting information


Table S1.

Table S2.

## Data Availability

The data supporting the findings of this study are available from the corresponding author upon reasonable request.
