# Supplementary material for: Understanding variations in the use of tranexamic acid in surgery: A qualitative interview study
Source: Br J Haematol. 2025 Feb 18;206(3):965–76. doi: 10.1111/bjh.20008 (PMC11886940; doi:10.1111/bjh.20008)
Supplement: Supplementary file 1 — Table S1. [file BJH-206-965-s001.docx]

Supplementary Table 1: Summary of domains and questions

| **Domain** | **Questions** |
| --- | --- |
| Behavioural regulation | Do you discuss TXA use with other professionals as part of this decision? Do you usually agree/disagree on whether TXA use is indicated? Why?  Do you estimate surgical blood loss or also that lost in the recovery period?  Do you have experience of electronic guided decision making for transfusion decisions like the administration of TXA? |
| Beliefs about capabilities | How confident are you/your colleagues about recommending, prescribing or administering TXA for individual patients?  How confident do you feel in decision-making around TXA use? What affects how confident you feel in using it? What would increase your confidence in using it?  Can you think of a case in which you were unsure as to whether to use TXA? Why? What happened? What did you do in the end? How often are you unsure as to whether to use TXA? |
| Beliefs about consequences | Do you think TXA is beneficial to patients? Are there any risks? Do the benefits outweigh the risks? What do you think about the evidence for TXA use?  What has been your experience of using TXA in practice?  Do you think specific groups of patients benefit more or less from the use of TXA?  Would you be comfortable to change a clinical decision because of a clinical decision support systems (CDSS) alert?  Do you think there is enough guidance on TXA use? Do you think others in your role are aware of the guidance? How could access to/knowledge of guidance be improved?  Do you believe there are limitations of the guidance (or areas of ambiguity) |
| Emotion | How does or would prescribing TXA make you feel (e.g. anxious, relieved, satisfied, concerned etc)  How do you feel about the use of CDSSs?  Do you or your colleagues have concerns about the use of TXA in surgery? If so what are you concerns? Does this apply to specific patient groups? |
| Environmental context and resources | Has the recent blood shortage driven changes in your practice around transfusions?  Is TXA use as part of the briefing/checklist in your organisation?  Is there enough training/education about the use of TXA? Would you want to know more about it before prescribing more frequently?  Is there anything about your specific environment which helps or hinders you prescribing of TXA?  What do you think might help support the use of TXA according to national and hospital guidelines?  What would improve uptake of TXA in practice do you think?  How implementation and any known barriers/enablers were identified and considered in development of the guidance |
| Knowledge | Do you believe the average blood loss is less than or greater than 500ml for each  How is TXA use decided for each patient?  What alternatives do you use?  Is TXA always available to you?  Are you aware of any guidance on use of TXA in your specialty?  How is TXA use documented at their institution (intravenous and local as it is often different for each route)?  Design rationale and evidence base for the guidance (including intended use) |
| Motivation and goals | Do you think that a CQUIN on the use of TXA in surgery encourage its use in compliance with national and hospital guidelines?  What do you think best drives changes in practice around transfusion medicine?  What support could be provided for someone in your role regarding appropriate TXA use? |
| Nature of the behaviour | Would inclusion of TXA in the WHO surgical checklist change its use? Is it already included in the checklist in your hospital?  Would electronic prompts (within the patients’ electronic records) change use?  Are there things you/your team/hospital etc need to do so that TXA is used more widely for surgical patients?  Who needs to do what differently when, where, how, how often and with whom to prescribe TXA (or prescribe it more often than at present)?  What procedural changes could be made to increase usage? Are there any changes around the usage of TXA that you would like to see? If so how could these be done? |
| Skills | Is it difficult to estimate total blood loss for your patients? |
| Social/professional role and identity | Could you tell me about your role and the team in which you work?  How long have you worked in this setting? Have you worked in similar settings previously?  What kind of procedures do you usually do/assist with?  To what extent is recommending, prescribing or administering TXA part of your professional role?  Can you give an example of a time you made a change to your clinical practice? What drove this change?  Can you think of any examples of successful practice change which might work to encourage discussion and usage around TXA?  How do you think we could encourage more discussion around the use of TXA?  How do you think we could change clinical practice around TXA usage?  Who do you think is ultimately responsibility for the decision on whether TXA is used? |
| Social influences | Has the recent blood shortage driven changes in the practice of those you work with?  Do people you work with use TXA regularly? How does that affect your decision to use TXA? Do they encourage or discourage you to use TXA?  Have you worked in other settings where TXA is used more or less? Why do you think that was? |
